# INCITE: A randomised trial comparing constraint induced movement therapy and bimanual training in children with congenital hemiplegia

**DOI:** 10.1186/1471-2377-10-4

**Published:** 2010-01-12

**Authors:** Roslyn Boyd, Leanne Sakzewski, Jenny Ziviani, David F Abbott, Radwa Badawy, Rose Gilmore, Kerry Provan, Jacques-Donald Tournier, Richard AL Macdonell, Graeme D Jackson

**Affiliations:** 1 Queensland Cerebral Palsy and Rehabilitation Research Centre, School of Medicine, The University of Queensland, Brisbane, Australia; 2 Brain Research Institute, Florey Neuroscience Institutes (Austin), Melbourne, Australia; 3 School of Health and Rehabilitation Sciences, The University of Queensland, St Lucia, Queensland, Australia; 4 Department of Medicine, The University of Melbourne, Victoria, Australia; 5 Department of Neurology, Austin Health, Heidelberg, Victoria, Australia; 6 Department of Radiology, The University of Melbourne, Victoria, Australia

## Abstract

**Background:**

Congenital hemiplegia is the most common form of cerebral palsy (CP) accounting for 1 in 1300 live births. These children have limitations in capacity to use the impaired upper limb and bimanual coordination deficits which impact on daily activities and participation in home, school and community life. There are currently two diverse intensive therapy approaches. Traditional therapy has adopted a bimanual approach (BIM training) and recently, constraint induced movement therapy (CIMT) has emerged as a promising unimanual approach. Uncertainty remains about the efficacy of these interventions and characteristics of best responders. This study aims to compare the efficacy of CIMT to BIM training to improve outcomes across the ICF for school children with congenital hemiplegia.

**Methods/Design:**

A matched pairs randomised comparison design will be used with children matched by age, gender, side of hemiplegia and level of upper limb function. Based on power calculations a sample size of 52 children (26 matched pairs) will be recruited. Children will be randomised within pairs to receive either CIMT or BIM training. Both interventions will use an intensive activity based day camp model, with groups receiving the same dosage of intervention delivered in the same environment (total 60 hours over 10 days). A novel circus theme will be used to enhance motivation. Groups will be compared at baseline, then at 3, 26 and 52 weeks following intervention. Severity of congenital hemiplegia will be classified according to brain structure (MRI and white matter fibre tracking), cortical excitability using Transcranial Magnetic Stimulation (TMS), functional use of the hand in everyday tasks (Manual Ability Classification System) and Gross Motor Function Classification System (GMFCS). Outcomes will address neurovascular changes (functional MRI, functional connectivity), and brain (re)organisation (TMS), body structure and function (range of motion, spasticity, strength and sensation), activity limitations (upper limb unimanual capacity and bimanual motor coordination), participation restrictions (in home, school and recreation), environmental (barriers and facilitators to participation) and quality of life.

**Discussion:**

This paper outlines the theoretical basis, study hypotheses and outcome measures for a matched pairs randomised trial comparing CIMT and BIM training to improve outcomes across the ICF.

**Trial Registration:**

ACTRN12609000912280

## Background

Cerebral palsy (CP) is the leading cause of childhood disability with an incidence of 1 in 500 live births[1]. Hemiplegia accounts for 35% (1 in 1300) of these children and upper limb (UL) involvement is usually more pronounced than the lower limb[2]. Management of long-term disability and the burden of care on both the health care system and families are substantial. Recently the financial cost of CP was estimated at Aus$1.47 billion with Aus$124.1 million attributed to direct program costs[3]. Families and individuals with CP accommodate approximately 43% of these costs and the various levels of government the remainder[3].

From an individual perspective, the impact of hemiplegia can be described using the International Classification of Functioning, Disability and Health (ICF)[4]. At the level of Body Structure and Function, children with hemiplegia can present with changes in brain structure and function (structural MRI, neurovascular changes with fMRI) resulting in impairments of spasticity, muscle length, sensation, and weakness. Limitations in activity performance are common in areas such as self-care, school and household related activities. Participation can be restricted in home, school and broader community life and may in turn impact on quality of life (QOL). Intervention is therefore paramount to minimise long term disability, and optimise functional independence, societal participation and long term career aspirations.

There are many models of intervention targeting deficits in hand and arm function that aim to reduce activity limitations for children with congenital hemiplegia. Historically, neurodevelopmental treatment approaches (NDT) have been used in an attempt to ameliorate impairments of body structure and function with the assumption that this would transfer to gains in activity performance[5]. However there has been little empirical evidence to support these approaches[6,7]. Current management of motor dysfunction includes task-oriented, functional therapy or motor learning approaches that collectively use repetitive practice of functional and goal directed tasks[8-10]. The framework of dynamic systems theory underpins some of these contemporary approaches[11,12]. This theory purports that spontaneous movement is generated as a product of the interaction of many systems. These systems include the child, the task and the environment and the movement is generated in the most efficient manner for a particular moment[12].

Constraint induced movement therapy (CIMT) is a relatively new intervention derived from the basic sciences[13]. It comprises applying a restraint to the unimpaired UL coupled with intensive training of unimanual skills in the impaired hemiplegic limb. This approach has been shown to improve UL task performance in adults following stroke with retention of effects at twelve months post intervention[14]. Modification of this approach for children with CP has followed, but until recently efficacy was limited to case reports and small prospective studies[10,15-20]. Two small RCTs have been performed with positive effects for amount of use and emerging behaviours[10,21]. A Cochrane review concluded that there was emerging evidence supporting CIMT for children with hemiplegia[22]. A further systematic review[7] that investigated all UL interventions including CIMT, NDT and contemporary upper limb therapy included three RCTs of CIMT[10,21,23]. Pooling of CIMT data for meta-analysis was not possible due to small sample sizes, variation in the type of restraint used, differing outcome measures (many of which had no reported validity or reliability for the population), varying intensity, and dosage of intervention made comparison across trials difficult[7]. Our systematic review concluded that evidence for CIMT should therefore be viewed cautiously and recommended that further suitably powered RCTs using valid and reliable outcome measures were required.

Conceptually, there are a number of key difficulties in the application of CIMT to children with CP. Children with hemiplegia, unlike adults who acquire a hemiplegia subsequent to a stroke, have never learned to use their UL effectively so demonstrate developmental disregard and/or non-learned use of their impaired limb[10,16]. Consideration of developmental aspects of acquiring UL skills and critical periods of brain development related to UL co-ordination are therefore important. Early bimanual use of both hands is thought to be important for the development of the assisting hand, as the development of motor control is modelled on effective use of the dominant hand[24]. In healthy adults, the contralateral and ipsilateral sensory motor cortex appear to act in a co-ordinated fashion during unilateral hand movements[25]. Constraint or reduced use of the dominant hand in young children may lead to activity dependent competitive displacement of the surviving contralateral corticospinal projections from the affected cortex by more active ipsilateral corticospinal projections from the affected motor cortex, thereby compounding the problem[26]. In addition, children with hemiplegia have bimanual coordination difficulties over and above their unimanual deficits[27]. The relationship between unimanual capacity and bimanual performance is not clearly understood and it is uncertain whether gains in unimanual capacity will transfer into improvements with bimanual performance. A discrepancy between unimanual capacity and bimanual performance is often observed in these children. When doing functional tasks such as putting on shoes and socks, children may adeptly use their unimpaired limb with little involvement of their impaired limb. However, when directed to do tasks with their hemiplegic limb they can demonstrate unimanual skills that are not utilised in bimanual tasks. Motor learning principles would suggest that improvement in use of two hands together will be maximised by repetitive practice of bimanual goal directed tasks[27].

Historically, therapists have focused on a bimanual approach (BIM training) in the management of motor dysfunction in children with hemiplegia[28]. Recently, a publication of intensive bimanual training, Hand Arm Bimanual Intensive Training (HABIT) was described by Charles and Gordon[27]. This approach focused on equal use of both hands in bimanual tasks and was developed in response to the limitations of CIMT to address bimanual co-ordination while maintaining the positive aspects of intensive training of the impaired limb[27]. A small RCT compared HABIT (n = 10) to a delayed treatment group (n = 10). Treatment intensity was equivalent to that employed in CIMT trials, for 6 hours per day over a 10-day period (total 60 hours training). A small treatment effect (η^2 ^of 0.256) favouring HABIT was achieved on a validated bimanual assessment the Assisting Hand Assessment (AHA)[29]. To date, CIMT and BIM training have not been directly compared in a randomised clinical trial. A further limitation of all previous studies of UL interventions has been the lack of standardisation of intensity or environmental context for the comparison or control treatments[7]. These parameters will be addressed in the present study.

The current study is funded by the National Health and Medical Research Council (NHMRC) of Australia (project grant 368500). The broad aim of this study is to evaluate in a single blind randomised matched pairs comparison trial whether a novel rehabilitation, CIMT, is more effective than BIM training to improve UL function, societal participation and QOL in children with congenital hemiplegia aged 5 to 16 years.

## Methods/Design

A matched pairs randomised trial will be conducted using an activity based day camp model[30] to evaluate the efficacy of CIMT compared to BIM training in children with congenital hemiplegia aged 5 to 16 years.

The specific hypotheses to be tested are:-

1. CIMT will reduce activity limitations and participation restrictions to a greater extent than BIM training in children with hemiplegia.

2. CIMT will result in greater use-dependent brain reorganisation compared to BIM training and this cortical plasticity will be retained for longer periods.

3. CIMT will result in greater enhancement of participation in school and community roles.

4. CIMT will result in greater improvements in QOL.

These hypotheses will address the following specific aims:-

1. Upper limb rehabilitation consumes a great deal of time and is costly, and the effects may be short lived. CIMT is a novel upper limb training where the unimpaired arm is constrained in a "glove like splint" while the impaired hemiplegic arm is intensively trained. This study will determine which training approach has the greatest impact on activity limitations and how long that effect is retained.

2. If one approach results in greater enhancement and retention of cortical reorganisation then this will guide clinical practice with implications for other patients (Traumatic Brain Injury TBI, stroke). An understanding of the nature and timing of the brain lesion may indicate which children respond better.

3. If a training program enhances community participation and improves QOL this will have a profound impact on educational opportunities and vocational roles.

**Figure 1:**
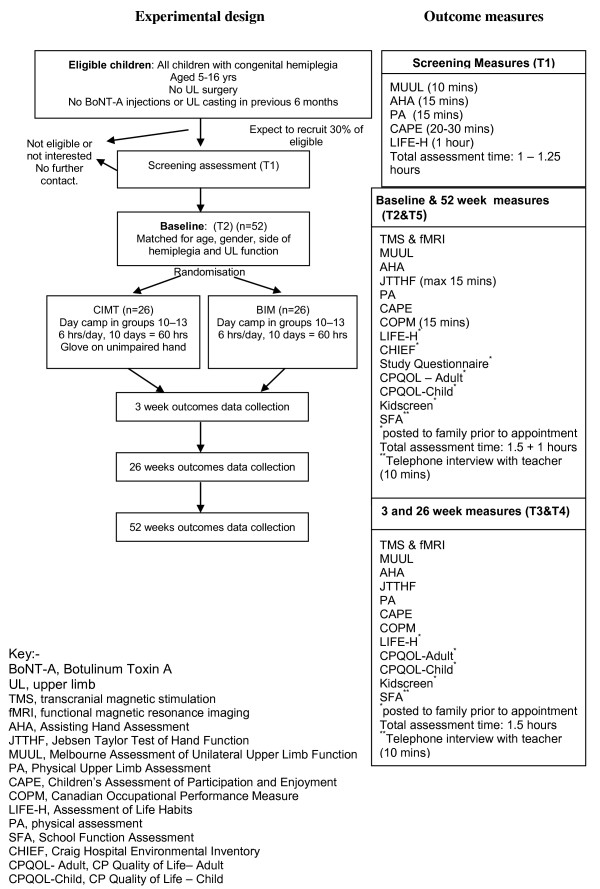
**Flow chart of INCITE study according to CONSORT guidelines**.

Assessments will be performed at baseline prior to the camp and then at three weeks following completion of the camp. Further follow-up will be performed at 26 and 52 weeks following intervention to determine retention of effects. The experimental design and outcome measures are depicted in Figure 1.

The research ethics boards at the Royal Children's Hospital, Melbourne (HREC 26074 A,B,C,D), La Trobe University (HEC 06-68), Royal Children's Hospital, Brisbane (HREC 2008/018) and The University of Queensland (2008000961) have granted approval for the study.

### Study sample and recruitment

Children and youth will be recruited from across Victoria and Queensland, Australia. The Australian health system allows all individuals access to publicly funded services and reimbursement for private medical specialists. The recruitment process will target both publicly funded services and private practitioners with the expectation that the sample will be representative of children with congenital hemiplegia. Furthermore, financial support for families from regional areas will be made available to allow equity of access to the program and a representative sample of children from metropolitan, outer metropolitan and rural/regional/remote areas.

### Inclusion criteria

The study will include children and youth:

1. With a confirmed diagnosis of congenital hemiplegia.

2. Aged 5 to 16 years.

3. With predominant spasticity rather than dystonia interfering with UL function according to the classification of motor type by Sanger et al[31] with Modified Ashworth Scale (MAS) grade >1 but <3[32].

4. Ability to achieve minimal active grasp with the impaired hand.

5. Sufficient co-operation and cognitive understanding to participate in the group activities.

For a subset of children performing the Advanced Brain Imaging and Transcranial Magnetic Stimulation (TMS) studies further inclusion criteria are:-

1. Sufficient co-operation to perform Advanced Brain Imaging studies for 45 minutes.

2. No exclusions for 3 tesla Magnetic Resonance Imaging (3T MRI) including no metal implants, no shunts, no uncontrolled epilepsy as the later would be a confound.

3. For TMS there must be no current or previous history of epilepsy.

### Exclusion criteria

1. Severe muscle spasticity and/or contracture (MAS > grade 3, i.e. muscle contracture or rigidity) which would require spasticity management or serial casting.

2. Previous orthopaedic surgery in the UL.

3. Serial casting or Botulinum Toxin A (BoNT-A) injections in the UL within 6 months prior to study entry.

### Sample size

According to CONSORT guidelines the primary basis for sample size calculation is adequate power for the comparison between the functional effects of CIMT and BIM training at 3 weeks post intervention. Based on data from a previous study[33], a mean difference of 7 percentage points was proposed or 10% of the anticipated control group mean at baseline on the Melbourne Assessment of Unilateral Upper Limb Function[34] as the minimum difference that is likely to have substantial clinical importance. Our pilot data in a study of UL training with and without intramuscular BoNT-A injections yielded standard deviation of changes of 7.6 and 9.6 units in the two groups. Based on a t-test comparison of changes using a SD of 9 units for both groups, significance (alpha) level of 0.05, and 80% power, we require 26 participants in each group (total sample of 52).

### Randomisation

Children will be matched in pairs according to age (12 month age bands), gender, side of hemiplegia, and level of functional ability based on MUUL scores at screening. Once matching has been achieved and baseline assessments completed, children will be randomised within pairs from concealed envelopes opened by non-study personnel. Treatment allocation will be recorded on a piece of folded paper inside each envelope in random order (computer generated). The randomisation process will involve allocating a number "1" or "2' to each member of the pair which will be written on the paper inside the envelope. As each pair is entered, they will be allocated the next consecutive envelope, which will be opened by the non-study personnel who will read and record the treatment allocation from the paper inside the envelope. Study personnel will be informed of group allocation.

A matched pairs design is the design of choice as it minimises the likelihood of group differences at baseline that has often been present in UL rehabilitation studies[21,29]. In order to maximise group coherence, camps will be conducted in two age bands (two camps each of CIMT and BIM training for children 5-10 years and one camp each of CIMT and BIM training for children 11-16 years).

### Study treatments

Each intervention will be delivered in groups of 9 to 13 children for 6 hours per day for 2 weeks (10 days) in a day camp model. The total dosage of intervention will be 60 hours. After baseline assessment and randomisation children will attend either the two week BIM training camp or immediately following, a two week CIMT camp. One week of the camp will be during school term and one during school holidays. To optimize children's engagement in the intervention, day camps will be run at a community sporting facility and will employ a circus theme with circus activities provided by professional trainers and an additional day-excursion to an adventure "low ropes" course. Consistent with self determination theory[35] to maximize motivation the key elements of competence, autonomy and relatedness will be incorporated. Competence will be achieved through scaffolding of activities to optimise success, autonomy by allowing choice, and relatedness through the sharing of the experience with others of similar abilities.

Both groups will receive the same dosage and content of intervention, delivered in the same environment. Participants in the CIMT group will wear a tailor made glove on their unimpaired limb while attending the day camp, which will only be removed for toileting. Table 1 summarises the components of each intervention. The tasks for each camp will be designed to enable either maximal bimanual use of the hands for the designated tasks and modified for the CIMT camp to perform the same tasks in a unimanual approach with constraint on the unimpaired hand.

**Table 1:** Summary of intervention content

Session (and Activities)	Allocated Time	CIMT: Type of involved hand use	BIM training: Type of involved hand use
**Fine motor activity stations**			
a) Games	1 hour	Reach, precision grasp, power grasp, tool use, controlled placement and release, finger isolation, in-hand manipulation, wrist extension, supination	Stabilisation, symmetric and asymmetric bilateral movements, precision grasp, controlled placement and release, finger isolation, in-hand manipulation, wrist extension, supination
b) Functional tasks			
c) Cooking			
d) Art and crafts			
**Circus Workshop**			
a) Warm up and stretch	2 hours	Active stretching whole body	Active stretching whole body
b) Ribbons		Grasp, shoulder external and internal rotation, adduction, horizontal abduction, flexion, elbow and wrist extension, supination	Grasp, shoulder external rotation, adduction, horizontal abduction, elbow and wrist extension. Symmetric movements.
c) Devil Sticks		Grasp, shoulder adduction and flexion, elbow and wrist extension	Grasp, symmetric shoulder flexion, elbow extension, wrist extension. Asymmetric shoulder adduction.
d) Hoops		Grasp, shoulder adduction and flexion, elbow extension, supination and wrist extension.	Grasp, shoulder adduction and flexion, elbow and wrist extension. Asymmetric movements
e) Spinning plates			Grasp, shoulder adduction and flexion, elbow extension, supination and wrist extension, finger isolation, asymmetric bilateral movement
f) Acrobalance		Strength, dynamic and static balance	Strength, dynamic and static balance
g) Aerials		Power grasp, upper body strength.	Power grasp, upper body strength & symmetry.
**Mealtimes**			
a) Preparation	1.5 hours	Grasp and transportation, precision grasp, tool use, elbow flexion, extension, supination, wrist extension, radial deviation	Stabilisation, grasp, tool use (knife and fork), asymmetric bilateral movements
b) Eating			
c) Cleanup			
**Games**			
a) Parachute		Grasp, shoulder flexion, extension, adduction, elbow and wrist extension, supination, throw and catch	Symmetric and asymmetric movements, stabilisation, grasp, shoulder flexion, extension, adduction, elbow and wrist extension, supination
b) Sports			
**Debriefing**	0.5 hour	Group discussion about day	Group discussion about day

Children will return to their regular therapy programs at the completion of the 2-week program. Documentation of concurrent therapy programs (ongoing, additional therapy or interventions, change in spasticity, medications or lower limb interventions) will be recorded at each follow up assessment as these would not be able to be controlled over a 12 month period.

### Therapy protocols and delivery

Three occupational therapists (LS,RG,KP) and one physiotherapist (RB) will plan and lead all intervention groups. The core therapists will be responsible for daily grading of activities and modification of tasks for the participants in each group as required. Planning activities for each group will require task analysis, and guidelines for grading to challenge children with varying capabilities. It is expected that many activities prepared for the BIM training group (focusing on bimanual tasks) will need to be modified for the CIMT group (focusing on unimanual tasks). An example of an activity analysis and grading for groups is provided in Additional file 1, Table S2. Volunteer occupational therapists, physiotherapists, human movement scientists, therapy students and sports recreation staff (YMCA) will assist with program delivery with a ratio of 2 participants to one staff member. Prior to commencement of the daily program, staff will be briefed and given specific tasks with written instructions outlining how each activity will be performed for the specific children they are supervising. Task content record sheets will be completed each day, summarising each activity for every child, including time taken, number of repetitions and how well the children performed the activity and their level of engagement. Video footage of each session will be taken for further independent content analysis. A debriefing session for the entire group with an independent staff member and separately for the staff will be conducted at the conclusion of each day.

Professional circus trainers will lead the two hour circus workshops and a YMCA leader and/or Human Movement scientist will run the gross motor games session. The core therapy team will meet with the circus trainers and gross motor program leader to design these programs and at the end of each session to discuss and modify the program as required and provide guidance to grading of tasks for participants. The core therapy team will also meet daily to review individual participants' goals and continually grade their program. A daily record of attendance will be kept.

### Outcome measures and procedures

A number of classification measurements will be used to describe the sample at the level of brain structure, brain reorganisation, manual abilities, severity of impairment (body structure) and gross motor function. This study will be the first to our knowledge to comprehensively measure and compare the impact of two models of UL intervention across all domains of the ICF[4].

#### 1. Classification of the sample

The participants entered into the study that meet the inclusion criteria will be classified according to:

##### a) Manual Abilities Classification System (MACS)

The MACS classifies the child's ability to handle objects in daily activities on one of 5 levels[36]. The MACs has reported construct validity, and excellent inter-rater reliability (ICC 0.97 between therapists and 0.96 between therapists and parents)[36]. It is expected that all children in the sample will be MACS level I (able to handle objects easily and successfully) or level II (able to handle most objects but with somewhat reduced quality and/or speed of achievement so that alternate ways of performance might be used).

##### b) Gross Motor Function Classification System (GMFCS)

The GMFCS classifies the child's ability to carry out self initiated movements related to sitting and walking across 5 levels[37]. The GMFCS has strong construct validity with the Gross Motor Function Measure (r = 0.91)[38] and good inter observer reliability between professionals and between professionals and parents[39,40]. In this sample of children with congenital hemiplegia it is expected that most children will be GMFCS level I or II.

##### c) Zancolli Scale

The Zancolli Scale[41] classifies severity of forearm alignment by measuring the contribution of spasticity and muscle length in the wrist and finger flexors in active wrist and finger extension. The Zancolli scale was developed to classify impairment and alignment of the hemiplegic hand before and after surgery. Three levels range from I (minimal flexion spasticity, complete extension of fingers with wrist in neutral position or less than twenty degrees of flexion) to III (severe flexion spasticity, no extension of fingers even with maximal wrist flexion). It is expected that participants will have either a level I or II Zancolli score.

##### d) House Functional Classification Scale

The House Functional Classification Scale[42] was originally developed to evaluate hand function following surgery. It consists of nine grades ranging from 0 (does not use) to 8 (full spontaneous use). The House scale will be used to rate functional use of the impaired UL. Both the Zancolli Scale and House Functional Classification Scale have been found useful in describing hand function in a population based survey of children with CP[43].

#### 2. Primary and Secondary Outcome Measures

The primary outcome measure of interest is UL activity performance for unimanual capacity using the Melbourne Unilateral Upper Limb Assessment at 26 weeks follow up. Secondary measures include measures across all domains of the International Classification of Functioning, Disability and Health (ICF).

Measures of body structure will include both the positive features of the upper motor neurone syndrome (muscle spasticity, length) and the negative features (weakness, dexterity and sensation). Positive features will be assessed in the impaired (hemiplegic UL) while negative features will be compared between limbs (to account for any unintended effects of wearing the constraint on the unimpaired hand in the CIMT group).

Measures of activity will assess unimanual capacity in the impaired limb (using the MUUL), bimanual performance between the upper limbs (using the Assisting Hand Assessment) and the achievement of individualised outcomes (with the Canadian Occupational Performance Measure). Participation will be assessed at home, in school and community life using both child and parent proxy response. Environmental barriers and facilitators to participation will be evaluated. In addition to these measures, the broad domains of quality of life will be assessed.

#### 3. Neurovascular measures

##### a) Whole-brain functional MRI studies

We have previously used functional Magnetic Resonance Imaging (fMRI) to localise the motor cortex in patients with tumours and dysplastic lesions[44], to monitor the sensory cortex in patients recovering from stroke[45,46] and to examine functional reorganisation in cerebral palsy[46,47] and have combined this with TMS to localise the motor cortex in stroke patients[48]. We now plan to use similar techniques to investigate brain reorganisation in response to therapy in children with CP. Brain reorganisation has been demonstrated using TMS and fMRI following intensive UL training in adults after chronic stroke[49]. Functional MRI or blood oxygen level dependent (BOLD) contrast is a robust and non-invasive method of detection of regional tissue changes in venous oxygenation in response to task related activation[50].

Functional MRI has provided new information in understanding real time cerebral blood flow changes in response to behavioural activation[51]. Recent fMRI studies have shown that individuals with chronic stroke can be trained to improve finger control with intensive practice and this can be accompanied by increases in motor cortex activation[49]. It has been suggested that intensive arm training may result in rapid brain reorganisation[52]. Our group has undertaken the first study of serial fMRI in young children with CP to determine central neurovascular responses to treatment[33]. We have achieved 90% compliance with serial fMRI and good reproducibility of the location of activation for the unimpaired limb for two motor tasks in children with hemiplegia. There are a very limited number of motor studies with fMRI in children with CP and no serial fMRI motor studies measuring outcome associated with intense models of UL training.

Functional imaging at 3 tesla on a Siemens MAGNETOM Trio MR scanner will be conducted at the Brain Research Institute, Melbourne. This research dedicated 3T scanner provides approximately twice the signal to noise ratio for functional imaging compared to conventional 1.5 tesla scanners. We take advantage of this to reduce the time in the scanner for children and improve the resolution of data collected. To prepare for the real fMRI scan all children will practice in a mock MRI scanner using techniques that have achieved 90% compliance in our earlier studies. During scanning of their anatomical images the children will be able to watch a favourite video. Children will lie supine, with their head immobilised with one immobilisation pad to minimise head movement. In the scanner, children will perform two motor tasks, active and passive wrist extension at 2 Hz. These tasks are frequently impaired in children with hemiplegia and most likely to show a response to training. Individual forearm resting splints will standardise the starting positions. The motor paradigm will consist of a 2-condition block design, visually cued via instructions projected on a screen. The baseline condition is no movement. A tape recording of a metronome at 2 Hz will provide an auditory cue for the rate of movement. Verbal cues to commence and end the task will be given. The task and rest periods are 30 seconds with the activation cycle repeated four times. An additional five minutes of resting-state fMRI will also be collected for analysis of functional connectivity. Tasks performed prior to resting-state fMRI can influence functional connectivity[53], so the resting-state data will be collected after the motor paradigms have been completed to provide some consistency in this regard. The whole assessment will take no longer than 45 minutes. The actual movements performed in the scanner will be rated for speed, the range of motion actually performed, ability to isolate the movement and presence of mirror movements in the contralateral hand or general body movements. If there is evidence of task-correlated breath-holding[54], the task will be repeated.

Functional MRI will be acquired using a BOLD acquisition sequence (Gradient-recalled-echo (GRE) echo-planar imaging (EPI), Repetition Time (TR) = 3.0 s, Echo Time (TE) = 30 ms, Flip angle = 85°, Slice thickness = 3 mm, FOV = 216 mm, 44 slices, 72 × 72 matrix yielding an in-plane resolution of 3.0 mm × 3.0 mm). A single set of T2-weighted anatomical, FLAIR and 3D T1 volumes will also be collected. Functional MRI image processing, analysis and visualisation will be performed using iBrain(tm) software[46] and SPM software (Wellcome Department of Imaging Neuroscience, London, UK).

Pre-processing of the fMRI images will include slice-timing correction using a temporal interpolation scheme to estimate the response at the time of commencement of each acquisition volume, motion correction (realignment) within session and nonlinear registration across sessions for each participant, and spatial normalisation to the standard Montreal Neurological Institute (MNI) template supplied with SPM. In the realignment step, images within a session will be aligned to a single target image within that time series to minimise the effects of participant motion between scans. The target selected by iBrain(tm) is the image whose within-brain centre-of-mass is located closest to the median of all images in that time series. Target images from each session of a subject will then be non-linearly spatially normalised to a subject-specific space in an iterative fashion to ensure unbiased registration of images across sessions; this step is designed to correct, as far as practicable, non-linear image distortions that may differ from session to session. The step will be undertaken within subject rather than directly to the standard template to maximise the fidelity of within-subject registration. The mean of the within subject registered images will then be spatially normalised to the standard MNI template. Because many participants have large lesions, spatial normalisation to the MNI template will be undertaken using only an affine transform. In practice, the image transformations derived in each step described above will first be combined and then applied in one step to minimise resampling artefact when writing the final images. The spatially normalised image data will be smoothed with an isotropic Gaussian kernel at least twice the voxel size to fulfil the assumptions of Gaussian random field theory (RFT).

Using the general linear model (GLM), statistical parametric maps will be computed for each session of each subject. Temporal autocorrelation will be modelled using a white noise and autoregressive AR(1) model within SPM. Motion correction parameters will be included as covariates of no interest. Details regarding the specific implementation of the GLM and RFT by SPM are available elsewhere[55]. Due to the heterogeneity in lesion location and size across participants, group analysis of intra-participant change in activation will be undertaken using a region of interest approach with the assistance of iBrain(tm) software.

##### b) Diffusion Imaging Acquisition and White Matter Fibre Tracking

Participants will be scanned using a 3T Seimens MAGNETOM Trio whole body MRI scanner located at the Brain Research Institute, Melbourne. In addition to a number of standard radiological scans (T1, T2, FLAIR and 1 mm isotropic MPRAGE structural scan), diffusion-weighted images suitable for tractography studies will be acquired using a fully optimised single-shot, spin-echo echo-planar diffusion sequence. The imaging parameters will be (54 axial slices, TR/TE 7200/110 ms, 2.3 mm isotropic resolution, acquisition matrix: 104 × 104, parallel imaging reduction factor of 2, 60 diffusion encoding directions with a *b *value of 3000 s mm^-2^). The total imaging time for this sequence is 9 minutes. Diffusion-weighted MR white-matter tractography will be undertaken in a manner robust to crossing fibres, using constrained spherical deconvolution (CSD) and probabilistic streamlines[56-58] using MRtrix software (Brain Research Institute, Melbourne Australia; http://www.brain.org.au/software).

##### c) Brain Reorganisation

Transcranial Magnetic Stimulation (TMS) will be performed on all participants in both groups at baseline then at 3 and 26 weeks post intervention. TMS will be delivered to both hemispheres. For each participant, care will be taken to perform both studies at approximately the same time of day so as to increase the repeatability of the measurements.

During TMS, participants will sit in a comfortable, reclining chair. Surface electromyographic (EMG) recording will be made from the abductor pollicis brevis muscle (APB) using disc electrodes in a tendon-belly arrangement. The motor evoked potentials (MEPs) will be recorded on an Oxford Medelec Synergy electromyography machine. Band-pass filtering (10 - 5000 Hz) was used. Sweep speed for threshold determination will be 100 ms and the gain will be set to 100 μV/div. Auditory EMG feedback will be given to ensure complete, voluntary relaxation of the target muscles.

The experimental session will record the following parameters:

###### i) Motor Threshold (MT)

Stimulation will commence at 30% of maximum output and increase in 5% increments until the motor evoked potential (MEP) is established. 1% changes in intensity will then used to calculate the threshold value. Motor threshold is defined as the lowest level of stimulus intensity which produced a MEP in the target muscle of peak-to-peak amplitude > 100 μV on 50% or more of 10 trials[59].

###### (ii) MEP Recruitment Curves

The maximum compound muscle action potential (CMAP) amplitude of the resting APB will be determined by supramaximal stimulation of the median nerve at the wrist. For each participant, the average of the CMAP amplitudes obtained after three stimuli will be calculated as was defined as 100%[60]. MEPs obtained by Single pulse TMS using different randomized stimulus intensities of 110, 120, 130, and 140% MT will be expressed as a percentage of the CMAP in order to obtain recruitment curves[61]. An average of 10 peak-to-peak MEPs recorded for each stimulus intensity will be calculated.

For motor thresholds and recruitment curve measurements, the stimulus will be delivered to the contralateral cerebral hemisphere using the appropriate direction of coil current flow (anticlockwise for left cortical stimulation and clockwise for right cortical stimulation). This will be performed using a flat circular 9 cm diameter magnetic coil (14 cm external diameter) connected to a Magstim stimulator (Magstim, Whitland, Dyfed, United Kingdom). The centre of the coil will be positioned over the vertex and held in a plane tangential to it. The coil will be held in place by a support stand, and its position will be checked regularly through each experiment.

###### (iii) Ipsilateral Motor Pathways

This will be performed using a figure-of-eight-shaped coil (outer diameter of each loop 70 mm) connected to a Magstim stimulator (Magstim, Whitland, Dyfed, United Kingdom). The coil will be placed tangentially over the ipsilateral hand motor cortex with the handle pointing back and laterally 45° away from the midline at the optimal site for the activation of the APB. This is thought to be the best position for activating the pyramidal cells trans-synaptically and preferentially elicits late I-waves[62]. The direction of current induced in the brain will be anterior to posterior.

#### 4. Body functions and structures

The positive features of the UMN syndrome will be measured to grade the impact of intervention on the positive impairments and to compare severity between the groups after random allocation. Active and passive range of motion will be assessed primarily for the impaired shoulder, elbow, forearm (pronator teres), wrist flexors (flexor carpi ulnaris, flexor carpi radialis), fingers and thumb adductors (adductor pollicis) using goniometry[33]. Spasticity will be measured using the Modified Tardieu Scale[63] at fast velocity in the forearm agonists and the MAS[32,64] in the same muscle groups.

The negative features of the UMN syndrome will be measured to describe the sample and grade the effects of intervention. Each test will be performed in both the impaired (hemiplegic limb) and unimpaired (hand writing) UL to compare sensory function between limbs and evaluate the effects of treatment on both limbs. Two aspects of sensory impairment will be measured.

(i) Stereognosis will be assessed on the impaired and unimpaired limbs using the approach originally described by Feys[65]. Three familiar objects (teaspoon, key, peg) and six similar matched objects (safety pin and paperclip; pen and pencil; coin and button) will be used. With vision occluded, children will be presented with each item. A corresponding set of items will be used to allow children to identify the object in order to minimise any errors due to incorrect naming of the object.

(ii) Moving two point discrimination (M2PD) will be measured using the Disk-criminator® (Baltimore, Maryland) on both the impaired and unimpaired limbs. Either one or two points will be randomly applied in continuous moving firm contact longitudinally to the pulp of the index finger with vision occluded[66]. The minimum distance participants can usually distinguish between two discrete points, ranging from 2 mm (normal) to 15 mm (poor) were recorded[67].

(iii) Grip strength will be measured using a hand held dynamometer (Smedley, Takei Scientific Instruments Co Ltd). Grip strength will be measured for three attempts on the impaired and unimpaired limbs (kilograms force, Kgf) according to the guidelines of the American Society of Hand Therapists[68]. The mean of the three attempts will be used to compare limbs and to evaluate changes over time.

#### 5. Activity Outcomes

##### a) Unimanual capacity

###### (i) Melbourne Unilateral Upper Limb Assessment of Function (MUUL)

The MUUL[34] measures aspects of upper limb impairment and quality of upper limb function and will be the primary outcome measure. It consists of sixteen criterion-referenced items examining aspects of reach, grasp, release and manipulation. Each item has a set of scoring criteria with maximum possible raw score of 122. Raw scores are computed into percentage scores. The MUUL test has very high internal consistency (α = 0.96)[69]. Inter-rater and intra-rater reliability is very high for total test scores (ICC 0.95 and 0.97 respectively) and moderate to high for individual items (ICC 0.69 - 0.91). Test-retest reliability is moderate to high for items and high for total test scores[69]. The MUUL has established construct and content validity during test development[34]. Comparison of the MUUL and components of the Pediatric Evaluation of Disability Inventory (PEDI) yielded high correlation coefficients, further supporting construct validity[70]. Previous results of a reliability study found a change of 12% for intra-rater reliability and 14% for inter-rater reliability was required to suggest a clinically significant effect[34]. In a subsequent study investigating the interrater reliability and measurement error of the MUUL in a group of children with hemiplegia aged 5 to 8 years, results yielded a standard error of measurement (SEM) of 2.6% with the smallest detectable difference (SDD) of 8.9%[71]. A further investigation of interrater reliability and SEM of trained and untrained raters yielded SEM of 2.56 and 3.37 respectively[72]. Together these studies suggest that the SDD may be smaller than that originally published by Randall et al. However, reliability studies differ in terms of the study population in relation to age and type of CP, making it difficult to generalise results to our study population assessed by one trained rater masked to group allocation. Establishment of intra-rater reliability for this study is therefore required to determine the SDD and define children who achieve a significant clinical response.

###### (ii) Jebsen Taylor Test of Hand Function (JTTHF)

The JTTHF measures unilateral speed and dexterity on timed tasks[73]. The test measures speed and accuracy of performance on various complexities of grasp and release tasks using everyday items. The original test designed and validated in adults and typically developing children will be modified with omission of the writing activity and by reducing the maximum allowable time of each task to 2 minutes to both reduce frustration and allow comparison with similar studies in children with congenital hemiplegia[21,29,74]. The JTTHF has been shown to be responsive to change due to an intervention, however there are difficulties with stability of test-retest performance in the unimpaired limb[21,29,30,74].

##### (b) Bimanual performance

###### Assisting Hand Assessment (AHA)

Bimanual performance will be assessed using the AHA. This is a Rasch analysed measure of the effectiveness with which a child with a unilateral impairment makes use of his/her impaired hand in bimanual tasks[75]. The test consists of twenty-two items that are videotaped and each scored on a four point rating scale, yielding a range of scores between 22 and 88. Scaled scores are calculated by transforming the total raw score to a percentage and range from 25 to 100. Rasch analysis allows conversion of these ordinal scores into logits (log odds probability units) which are equal interval measures. Inter-rater reliability is high for summed scores (ICC 0.98) as is intra-rater reliability (ICC 0.99). There are three versions of the AHA; small kids, school kids and an adolescent version. Test-retest reliability is high for small kids (ICC 0.99) and school kids (ICC 0.98) and reliability between the two forms (small kids versus school kids) is also high (ICC 0.99). The AHA has been shown to be responsive to change due to an UL intervention[29,30]. Investigation of reliability yielded a SDD of 3.89 raw scores for the small kids and 3.65 raw scores for the school kids version[76]. The AHA requires standardised training and certification of raters[75]. The AHA will be scored by one certified rater whom will be masked to group allocation and order of assessment.

##### (c) Individualised outcomes

###### Canadian Occupational Performance Measure (COPM)

The COPM[77] is a standardised individualised, client centred measure that evaluates client's self perception of occupational performance over time. Client's identify areas of difficulty in everyday occupational performance and rate their performance and satisfaction for each problem on a scale from 1 to 10. An average score for performance and satisfaction is calculated. The COPM was designed for all ages and disability groups. There is good evidence of construct, content and criterion validity[78-80]. The retest reliability of the performance and satisfaction scores on the COPM is high (ICC 0.76-0.89)[81,82]. The COPM has demonstrated responsiveness to change in paediatric clinical trials[83,84]. A 2 point change on COPM performance has been reported as being clinically significant[77] In the present study the COPM will form the basis of goal setting for therapy. The COPM will be administered by one of the study occupational therapists with the child/adolescent and where necessary with parental input.

#### 6. Participation Outcomes

Three assessments were chosen to capture participation restrictions across home, school and community life based on a systematic review of participation measures for children with hemiplegia[85].

##### a) Assessment of Life Habits (LIFE-H for children version 1.0)

The LIFE-H is designed for children aged 5 to 13 years and measures life habits in home, school and neighbourhood environments[86,87]. It is a questionnaire completed by the parent/caregiver about the child. The child form is based on an adult version[86]. The long form consists of 197 items divided into 12 categories and includes regular activities (eating meals, communication, and mobility) and social roles. A weighted score ranging from 0 to 10 is generated for each category and overall total. Evidence of construct validity was established during test development[87] and criterion validity with strong correlations between the LIFE-H and PEDI and Functional Independence Measure for Children (WeeFIM)[88]. Adequate to excellent internal consistency (α 0.73 - 0.90 for categories, 0.97 for daily activities and 0.90 for social roles), intra-rater (ICC 0.83 - 0.95 for daily activities), inter-rater (ICC 0.8 - 0.91 for daily activities and 0.63 - 0.9 for social roles) and test-retest reliability (ICC 0.73 for total score) have been established[89]. Four categories will be evaluated in this study including nutrition (eg. mealtime activities), personal care (eg. dressing), education and recreation. These areas were considered to reflect many of the identified difficulties confronted by children with congenital hemiplegia that might be amenable to the intervention program.

##### (b) Children's Assessment of Participation and Enjoyment (CAPE)

The CAPE was designed to measure childrens participation in formal and informal leisure and recreation activities outside of school[90]. It evaluates five aspects of participation, including diversity (number of activities participated in), intensity (frequency of participation), with whom, where (the environment) and the level of enjoyment. Scores can be generated for formal and informal domains and five activity types. Total test diversity scores can range from 0 to 55 and intensity scores from 1 to 7. These are the two areas of interest in this study. Content and construct validity were established during test development[90] and in a subsequent validation study[91]. Test-retest reliability is adequate for diversity (ICC 0.67-0.77) and intensity (0.72-0.81) and high for inter-rater reliability (0.82-0.99)[90]. The interview method of administration will be used, with the expectation of establishing rapport with the children in the study.

##### (c) School Function Assessment (SFA)

The SFA evaluates a child's performance in a range of functional tasks that are necessary for participation in academic and social activities in primary school[92]. The first section of the assessment specifically measures participation in six school activity settings (general or special education classroom, playground, transportation to and from school, bathroom, transitions to and from the classroom and mealtimes). The remaining two sections measure activity performance and task supports. All three sections can be administered independent of each other,[92] so in order to reduce burden, only the participation domain will be used. The SFA has established validity using expert panels and factor analysis[92,93]. The SFA has high internal consistency (Cronbachs alpha (α) 0.92 - 0.98 for scales), excellent test-retest reliability (ICC 0.80 - 0.99) and adequate interrater reliability (ICC 0.70 for participation)[92,94]. The participation section will be completed by the child's teacher during a telephone interview.

#### 7. Environmental Measures

##### (a) The Craig Hospital Environmental Factors (CHIEF)

This is a generic measure that evaluates the impact of attitude and support barriers, services and assistance barriers, physical and structural barriers, policy barrier, and work and school barriers to participation. It has excellent established reliability with high test-retest (ICC 0.93 for total score) and internal consistency (α = 0.93) and good content and discriminant validity[95].

##### (b) The Study Questionnaire

A study questionnaire was developed to capture demographic information that has been shown in the literature to influence a child's participation. These include family ethnicity, socio-economic status, family structure and supports, and family interests. A measure of social advantage/disadvantage will be derived from postcode of residence using the Index of Relative Socio-economic Advantage/disadvantage (2006) from the Australian Bureau of Statistics[96]. Deciles will be reported on a continuum with lower scores reflecting greater socio-economic disadvantage and higher scores reflecting socio-economic advantage.

#### 8. Quality of Life

##### (a) The Cerebral Palsy Quality of Life Questionnaire for Children (CPQOL-Child)

The CPQOL-Child[97] will be used to capture parental report of their child's perceptions of quality of life (CPQOL Primary Caregiver Questionnaire). For children and youth of nine years or older, the CPQOL Child Report Questionnaire will also be used to measure children's own perceptions of their quality of life. Results of factor analysis demonstrated that the CPQOL-Child measures 7 broad domains of quality of life: social wellbeing and acceptance, functioning, participation and physical health, emotional wellbeing, access to services, pain and impact of disability and family health. The internal consistency ranges from 0.74-0.92 and 2 week test re-test reliability ranges from 0.76-0.89. As expected, the domains moderately correlated with the CHQ, KIDSCREEN, and GMFCS. The CPQOL-Child is designed to be used to evaluate the effectivness of interventions for children with cerebral palsy and to gain further understanding about the determinants of QOL. The CPQOL-teen version for youth 14-18 years was not available at the commencement of the present study so that the child version was used for the entire sample. A recent publication has confirmed that the domains for the Teen version are very similair to the Child version[98].

##### (b) Kidscreen

In addition to the condition specific QOL tool, the CPQOL-child, a generic measure will be used to compare parents report of QOL for children with CP compared to their age, gender and environmentally matched peers (the buddies). The Kidscreen has been described as the most useful generic measure of QOL of children as it addressed the multidimensional construct of QOL through various domains and focused specifically on the well-being of children, as the defined by the WHO definition of QOL[99]. It was developed to implement the views of children, through focus group work of 3000 children[100]. The questionnaire takes only 15-20 minutes to complete. The KIDSCREEN-52 Questionnaire consists of 52 questions across 10 domains. These domains include 1) Physical Well-being, 2) Psychological Well-being, 3) Moods and Emotions, 4) Self-Perception, 5) Autonomy, 6) Parent Relations and Home Life, 7) Social Support and Peers, 8) School Environment, 9) Social Acceptance (Bullying), 10) Financial Resources. A child self report was administered for children 8-18 years old and a parent proxy version was administered for parents of children 5-18 years. Children below the age of 8 were unable to self-report as their reports were likely to be unreliable. Reliability was calculated by Chronbach's Alphas and ranged between 0.76 and 0.89 throughout the 10 domains of HRQOL. Convergent and discriminant validity were tested using information of child's physical and mental health. Correlations of up to 0.55 were found when correlating the KIDSCREEN-52 dimensions with frequency of physical complaints[100].

### Comparison to Typically developing children

Fifty-two typically developing peers ("buddies") matched with the study participants according to age, gender and classroom that they attend will be invited to participate. Typically developing children in this 'bring a buddy" system will be assessed at baseline, 26 and 52 weeks to provide a reference for participation in home, school and community life and QOL. At study entry the typically developing age and gender matched controls "the Buddies" will meet the following criteria:

### Inclusion Criteria

1. Typical development with no history of upper limb dysfunction, developmental or learning disability.

### Exclusion Criteria

1. Diagnosis of learning disability, developmental disability, history of behavioural problems, visual or hearing difficulties that would impact on function and participation.

2. History of congenital or acquired upper limb dysfunction.

The procedure for recruiting the "buddies" is that they will be identified by the study participants and their families, and will be in the same class at school and be invited to participate by the child with hemiplegia in the study. Once "buddy" participants agree to be contacted by the researchers they will be sent an information statement and consent form, followed by telephone contact to arrange time for an initial assessment. At the initial assessment the study and assessment procedure will be explained and the "buddy" will be screened. Each buddy will be assessed on the CAPE, LIFE-H, and Kidscreen to provide comparison data for the study sample. Only children attending regular school will be invited to participate to provide an age, gender and environmentally matched reference sample.

### Analyses

All assessment forms will be developed using scannable Teleforms (Teleform, 2005). Forms will be entered, scanned and visually checked by staff trained in use of the software. Data will be transferred electronically into Microsoft Access 1997. Analyses will be on an intention-to-treat basis using STATA 10. Data from each outcome measure will be summarised for each treatment group and descriptive statistics (frequencies, means, medians, 95% confidence intervals) calculated dependent on data distribution. We anticipate that groups will be similar on baseline measures due to the matched pairs design. Initially between group differences will be evaluated at the three follow-up assessment points using independent t-tests or Wilcoxon rank sum test. Within group changes between baseline and the follow-up assessments for continuous variables will be evaluated using paired t-tests or Wilcoxon matched-pairs sign rank test. A significance level of 0.05 will be used.

For the TMS data Student's paired t-test will be used for analysis of the difference between pre and each post intervention (3 and 26 weeks) mean MT and MEP ratios. This will be performed in each group for the impaired side and the unimpaired side.

To determine the intra-rater reliability of our trained occupational therapist rater on the MUUL, intraclass correlation coefficients will be calculated (3.1) with 95% confidence intervals from total MUUL percentage scores[101]. The standard error of measurement (SEM = SD × √(1-ICC)) and the smallest detectible difference (SDD = SEM × 1.96 × √2) will be calculated[102].

Post hoc analyses will be undertaken to investigate clinical characteristics of children who have a greater response to either intervention.

## Discussion

This paper presents the background and design for a matched pairs randomised trial comparing CIMT and BIM training for children with congenital hemiplegia. This study is the first to directly compare the two approaches and will be the largest study of either approach to date. Furthermore, we will be evaluating the outcomes of the intervention program across all domains of the ICF using valid and reliable measurement tools. It is anticipated that the results of this study will be disseminated widely through peer reviewed journals and international academic conferences.

## Abbreviations

CP: Cerebral palsy; CIMT: Constraint induced movement therapy; BIM training: Bimanual training; UL: Upper limb; MUUL: Melbourne Assessment of Unilateral Upper Limb Function; AHA: Assisting Hand Assessment; JTTHF: Jebsen Taylor Test of Hand Function; COPM: Canadian Occupational Performance Measure; LIFE-H: Assessment of Life Habits; CAPE: Children's Assessment of Participation and Enjoyment; SFA: School Function Assessment; CHIEF: Craig Hospital Inventory of Environmental Factors; CPQOL-Child: Cerebral Palsy Quality of Life Questionnaire for Children; TMS: Transcranial Magnetic Stimulation; fMRI: Functional Magnetic Resonance Imaging; ICF: International Classification of Functioning, Disability and Health.

## Competing interests

The authors declare that they have no competing interests.

## Authors' contributions

RB is the chief investigator and together with DA, RM and GJ designed and established this research study. LS and JZ also contributed to study design and RB, LS and JZ were responsible for the particular therapy contents. LS, RB and JZ were responsible for ethics applications and reporting. LS, RB, RG, and KP were responsible for recruitment, data collection, implementation of the studies in Victoria (LS, RB, RG) and Queensland (LS, RB, KP, JZ). DA, RB, GJ, and DT were responsible for the design, implementation, data collection, analysis of the Advanced Brain Imaging studies. RM, NB, DA and RB were responsible for the design and conduct of the Transcranial Magnetic Stimulation studies. LS, RB and JZ will take lead roles on preparation of publications on the clinical outcomes of the study and RB, DA, RM, NB, and GJ will take lead roles on the neuroscience publications from the study. All authors have read and approved the final manuscript.

## Pre-publication history

The pre-publication history for this paper can be accessed here:

http://www.biomedcentral.com/1471-2377/10/4/prepub

## Supplementary Material

Additional file 1 Examples of planning and modifications to fine motor activities for the Constraint Induced Movement Therapy (CIMT) and Bimanual training (BIMAN) groups. Click here for file
