# Supplementary material for: INCITE: A randomised trial comparing constraint induced movement therapy and bimanual training in children with congenital hemiplegia
Source: BMC Neurol. 2010 Jan 12;10:4. doi: 10.1186/1471-2377-10-4 (PMC2832893; doi:10.1186/1471-2377-10-4)
Supplement: Additional file 1 — Examples of planning and modifications to fine motor activities for the Constraint Induced Movement Therapy (CIMT) and Bimanual training (BIMAN) groups. [file 1471-2377-10-4-S1.PDF]

TABLE 2 **Examples of planning and modifications to fine motor activities for CIMT and BIM groups.**

ACTIVITY	TASKS	UL GOALS	GRADING	MODIFICATION for BIM	MODIFICATION for CIMT
Making Grass heads	• wet stocking<br>• fill end of stocking with soil and grass seeds<br>• tie/seal end<br>• decorate pot<br>• create face on stocking<br>• assemble<br>• water daily<br>• cut grass with scissors	▪ supination (hold soil)<br>▪ gross grasp (pot, funnel, cup)<br>▪ fine grasp(seeds)<br>▪ tool use (scissors, spoon, scoop)<br>▪ finger strength<br>▪ ↑ in-hand manipulation	• squirter bottle versus pipette for watering- spoon/scoop/fingers for soil & seeds<br>• pre-cut craft items to decorate face & pot<br>• complexity of decoration<br>• stocking tied in knot or rubber band	• stocking held in supinated hand, soil & seeds transferred with other hand<br>• stocking closed with rubber band<br>• cut excess stocking with scissors<br>• cut shapes from felt/paper using scissors<br>• pot of glue with brush or gluestick with lid<br>• hold pot while watering<br>• seeds, craft items etc in small containers with lids to be unscrewed, opened /closed	• stocking placed over cup, hand used to scoop/transfer soil & seeds<br>• therapist hold stocking while child twist & cut<br>• glue in bowl, dip craft items & press onto face/pot<br>• stickers to decorate<br>• pot on table while watering
Ribbon sticks	• screw hook into end of dowel<br>• attach swivel to hook<br>• tear fabric into long strip<br>• thread fabric into swivel & tie knot	• fine grasp (swivel & hook)<br>• ↑ supination (screwing hook, wind up fabric)<br>• tool use (pliers, scissors)	• prepare/pre-assemble parts of activity<br>• built up handle over dowel<br>• ↑ speed, range, difficulty of UL	• maintain grasp and extend both arms to tear fabric<br>• swap hands during dance movements<br>• spin dowel with both hands to wind up	• tear fabric with partner/therapist<br>• dowel stabilized on table<br>• focus on unimanual actions with completed ribbon stick

	• dance/stretches/ full upper limb ROM activities with completed stick<br>• wind up fabric onto stick	• grip strength & sustained grasp<br>• shoulder abduction, elbow ext, wrist ext<br>• ↑ UL ROM all joints	dance movements		
Making Juggling balls	• pump up balloons to stretch<br>• put rice into balloon using funnel<br>• tie knot<br>• cover with 3 or 4 other balloons stretched over<br>• snip holes in balloons to make circles<br>• throwing/catching/ juggling activities	• UL strength/grasp (balloon pump)<br>• ↑ supination (hold funnel)<br>• tool use (spoon/scoop/cup to transfer rice bowl to funnel)<br>• in-hand manipulation<br>• scissor skills<br>• finger strength<br>• UL coordination<br>• grasp/release	• hand over hand support<br>↑ height of funnel, containers to ↑wrist ext & shoulder flex<br>• large/small pump size<br>• balloon size<br>• speed of task<br>• difficulty of juggling tasks<br>• completed no. of balls	• 2 handed pump<br>• minimal assistance<br>• bilateral juggling tasks<br>• stretch balloons with fingers	• work in pairs<br>• precut balloons<br>• rest funnel in cup<br>• one hand catch/throw activities<br>• one-handed pump
Chocolate Game	• Group game – throw a dice, if 6 have a turn<br>• go to table in centre, put on shirt, cut off one square of chocolate and eat, continue until another 6 thrown	• grasp/release/ manipulation of die<br>• dressing shirt<br>• cutting/cutlery use<br>• FM speed and planning	• fast group activity, no modifications during game	• shirt with buttons<br>• knife and fork to cut chocolate	• stretch fabric shirt<br>• pizza cutter for one hand cutting of chocolate<br>• non slip mat
Paper planes	• folding paper sheets<br>• copying model or instructions	• isolated finger extension to smooth folds<br>• shoulder, elbow, wrist	• ↑ difficulty of design<br>• hand over hand support	• stabilizing paper while folding<br>• incorporate cutting of paper to start size	• large pieces of paper<br>• focus on activities with completed planes i.e. grasp, throw

		ext to throw planes		• throw either hand	
Speed disc	• grasp one handle either hand<br>• separate hands quickly to send disc along string to partner	• maintain grasp with each hand<br>• horizontally abduct shoulders symmetrically then return to adducted position<br>• fully extend both elbows<br>• increase speed<br>• coordinate & anticipate the movement	• hand over hand with therapist<br>• ↑speed<br>• ↑ time on task	• build up handle to facilitate grasp with affected hand if necessary	• activity not suitable
Grip ball	• catching tennis sized ball on velcro covered paddle	• catching, throwing skills<br>• grasp/release<br>• grip strength to separate ball & paddle<br>• UL coordination<br>• finger extension & wrist extension to hold paddle	• ↑ distance to throw/catch<br>• ↑ speed, difficulty<br>• pulling ball off paddle<br>• velcro glove versus paddle<br>• ↓ball size	• swap throwing & catching hands	• only catch **or** throw, work with partner/therapist<br>• pull ball off paddle of partner
Cooking - Ginger Balls	• open containers<br>• scoop/measure ingredients<br>• mix<br>• melt in microwave<br>• shape<br>• cut baking paper	• UL strength<br>• tool use<br>• supination, wrist ext<br>• range of grasp, grip strength	• variable size spoons/scoops<br>• breakdown steps<br>• pinch & sprinkle coconut versus spoon	• ingredients in range of containers with lids<br>• rolling pin to crush biscuits<br>• tear baking paper on roll<br>• hold bowl, spoon,	• ingredients in open bowls from start<br>• hammer to crush biscuits<br>• pre-cut baking paper<br>• use heavy bowls & non-slip mats

	• set in fridge			ingredients off table<br>• build up tool handles<br>• place on large tray<br>• roll balls between hands	• work in pairs<br>• spoon into egg ring & flatten (not roll in hands)<br>• small containers to transfer
Cooking – Apple slinky	• put whole apple onto gadget, turn handle to slice into slinky	• sustained grasp<br>• alternating movement<br>• wrist ext		• hold gadget to stabilize with one hand, turn handle with other	• use suction &/or adult to stabilize gadget
Board games	• Jenga<br>• Connect 4<br>• Hyperdash<br>• Bop-it<br>• Speed Stack<br>• Don’t Panic<br>• Memory<br>• Stacking Glasses	• variety grasp size,shape,weight<br>• controlled release<br>• ↑UL speed<br>• ↑supination<br>• ↑active ROM<br>• UL coordination<br>• in-hand manipulation skills	• size or number of pieces<br>• speed, timed games<br>• ↑height of table or game<br>• hand versus finger to press buttons	• swap hands during game<br>• time both hands	• work in pairs<br>• larger pieces/cards<br>• non-slip mat
In-Hand Manipulation Activities	• Knuckles<br>• Frogs<br>• Play dough<br>• Magic<br>• String Hand Art<br>• Turning coins<br>• Paper clip chains<br>• Pen walks<br>• Lego/ construction<br>• Pop Beads	• in-hand manipulation<br>• fine grasp<br>• finger strength<br>• finger isolation<br>• supination	• size, number, complexity<br>• timed tasks	• transfer hand to hand<br>• time R&L	• one handed
Ball Activities	• Skittles<br>• Tray deflection<br>• Bat & ball	• ↑active ROM<br>• ↑response speed<br>• ↑UL coordination	• ↓size of ball<br>• size of bats<br>• vary speed,	• large balls for 2 hand catch<br>• 2 paddles/bats	• small balls<br>• tray with handle

	• Bounce/ catch<br>• Shoot hoops<br>• Ball / wall<br>• Foot launcher<br>• Frisbee<br>• Balloon tennis	• grasp/release<br>• grip strength	distance, height<br>• hit versus catch<br>• self versus in pairs, teams	• swap hands<br>• tray requiring 2 hands	
Craft Activities	• Jewellery making<br>• Threading<br>• Card making<br>• Door Hangers<br>• Rockets<br>• Kites<br>• Bindees<br>• Puppet making	• fine coordination<br>• in hand manipulation<br>• finger isolation<br>• grasp strength<br>• tool use<br>• ↑ROM	• complexity of task<br>• size of pieces/beads/cards<br>• quality &quantity of completed task<br>• positioning of pieces, tools	• lids on containers<br>• glue stick / brush & bottle<br>• cut with scissors	• pre-cut craft items<br>• partial pre-assembly<br>• non slip mat, stabilize on table<br>• work in pairs<br>• open containers<br>• glue in bowls to dip
Meal time Clean -up	• collecting dirty plates, etc<br>• filling wash up containers with water<br>• washing up<br>• drying dishes<br>• wiping table	• ↑ADL independence<br>• ↑ hand function	• expectations and amount of assistance	• large tray to carry plates<br>• hold utensils while washing , wiping<br>• wipe table with sponge while spraying cleaner	• use bucket with handle to carry plates<br>• work in pairs<br>• place utensils on table while wiping<br>• fill wash up bucket using jugs
